# Prostate cancer misdiagnosed as prostatic abscess: case report and literature review

**DOI:** 10.1186/s12894-025-01982-6

**Published:** 2025-11-18

**Authors:** Yanan Wang, Junguang Wang

**Affiliations:** Ningbo Yinzhou No.2 Hospital, Ningbo, 315192 Zhejiang China

**Keywords:** Prostate cancer, Prostatic abscess, Misdiagnosis, Diagnosis, Case report

## Abstract

**Background:**

As one of the most common malignant tumors in men, prostate cancer (PCa) has garnered substantial research investment from the medical community. However, due to multiple challenges in diagnostic techniques and clinical practice, cases of missed diagnosis and misdiagnosis still persist.

**Case presentation:**

This article presents a case of a 36-year-old male renal transplant recipient (RTR) who was initially misdiagnosed with prostatic abscess (PA) due to perineal discomfort, but was later confirmed to have PCa through surgery.

**Conclusion:**

This case highlights the similarities in symptoms and imaging manifestations between PCa and PA, as well as the complexity involved in diagnosing PCa. Furthermore, the article particularly underscores the significance of being vigilant about the increased risk of PCa following kidney transplantation, employing clinical examination methods judiciously, and enhancing the ability to differentiate PCa.

**Supplementary Information:**

The online version contains supplementary material available at 10.1186/s12894-025-01982-6.

## Background

Prostate cancer (PCa) is the most common malignant tumor in the male genitourinary system. According to the latest GLOBOCAN statistics from the World Health Organization in 2018, PCa ranks second in terms of incidence among male malignancies worldwide, second only to lung cancer, and is also the fifth leading cause of cancer-related deaths [1].With global aging and the continuous increase in life expectancy, the burden of PCa on public healthcare systems will keep rising. PCa typically manifests with urinary symptoms, such as frequency, urgency, nocturia, and hematuria. In the advanced stages of disease progression, patients may present with perineal distension and tenesmus. These symptoms partially overlap with those of conditions such as prostatitis and prostatic abscess (PA), which often leads to delays in disease treatment [2].

Here, we present a case of a 36-year-old renal transplant recipient (RTR) who had been experiencing pelvic pain for nearly one year. Preoperative multiple imaging examinations indicated PA, but postoperative pathology confirmed PCa. This rare case prominently demonstrates the significance of incorporating PCa into the scope of diagnostic considerations when diagnosing prostatic lesions in young patients.

## Case presentation

**Fig. 1:**
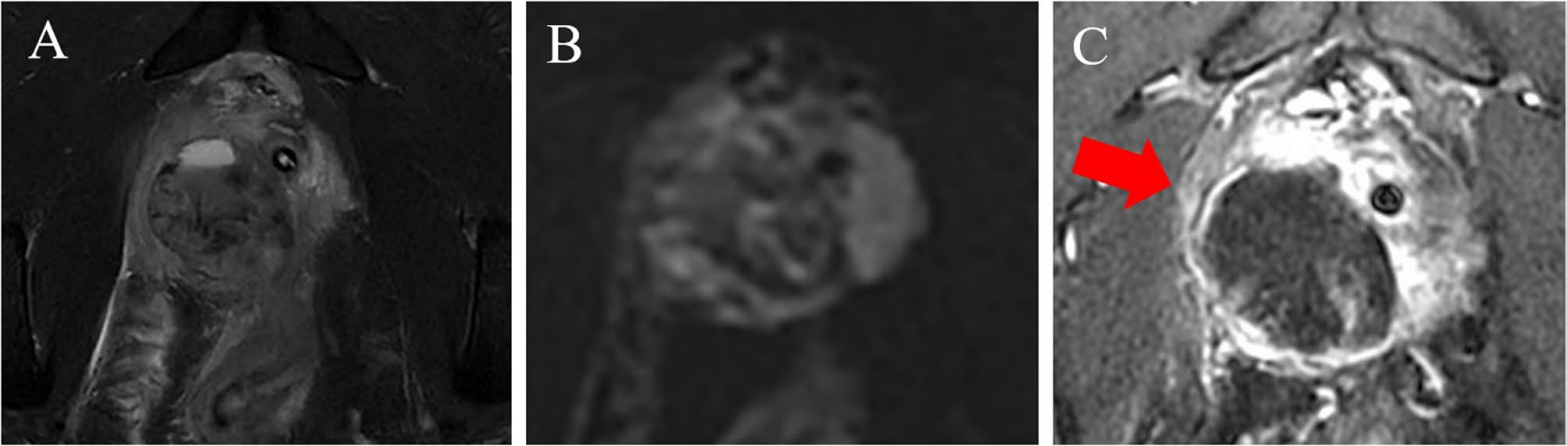
The boundary between the lesion and the posterior wall of the rectum is indistinct, with mixed signal intensity on T2WI-FS images (**A**). There is no obvious restricted diffusion (**B**), and patchy enhancement is observed within this region (red arrow, **C**)

**Fig. 2:**
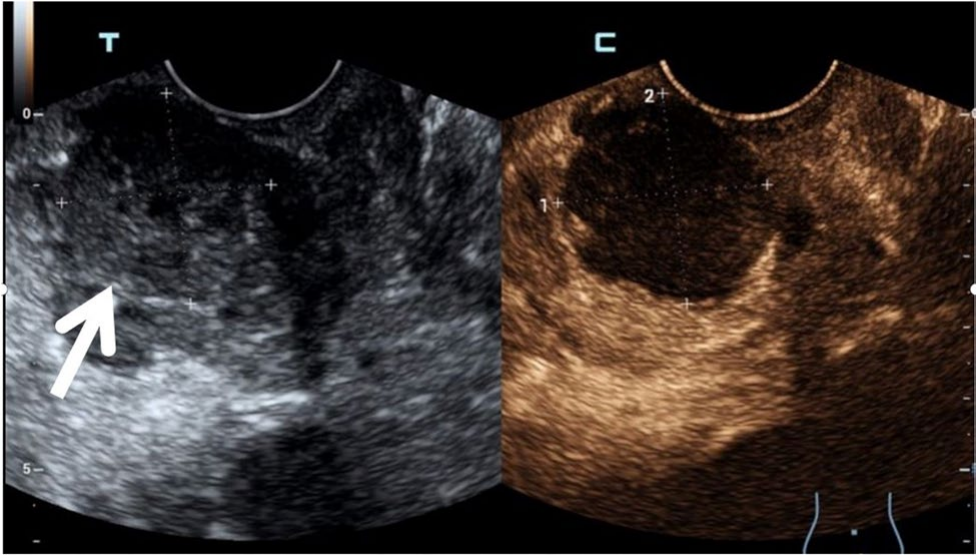
CUES reveals an inhomogeneous hypoechoic mass in the right lateral glandular region of the prostate (white arrow). After contrast agent injection, no significant contrast agent filling is observed in this region

A 36-year-old male presented to the hospital for medical treatment owing to perineal pain lasting for 7 months, with aggravation over the past 2 days. The patient underwent a kidney transplant 15 years ago and has been on long-term immunosuppressive therapy. After admission, the patient’s body temperature was 37.2 °C. A tender spot was palpable at the 11 o’clock position during digital rectal examination (DRE). Blood tests showed a white blood cell (WBC) count of 11.9 × 10^9/L, a C-reactive protein (CRP) level of 10.4 mg/L, a total prostate-specific antigen (tPSA) level of 4.53 ng/mL, and a serum creatinine (SCr) level of 121 µmol/L. Magnetic resonance imaging (MRI) of anal canal examination revealed abnormal signal foci in the prostate involving the seminal vesicles and rectum (Fig. 1A-C). Contrast-enhanced ultrasound (CEUS) suggested an unliquefied PA (Fig. 2). Based on a comprehensive evaluation of the aforementioned clinical symptoms and examination results, the patient’s preliminary diagnoses were: (1) Prostatic abscess; (2) Renal allograft dysfunction. Subsequently, the patient was administered cefoperazone-sulbactam for anti-infective therapy, along with tamsulosin hydrochloride sustained-release capsules to alleviate symptoms. One week later, the patient’s symptoms had alleviated.

**Fig. 3:**
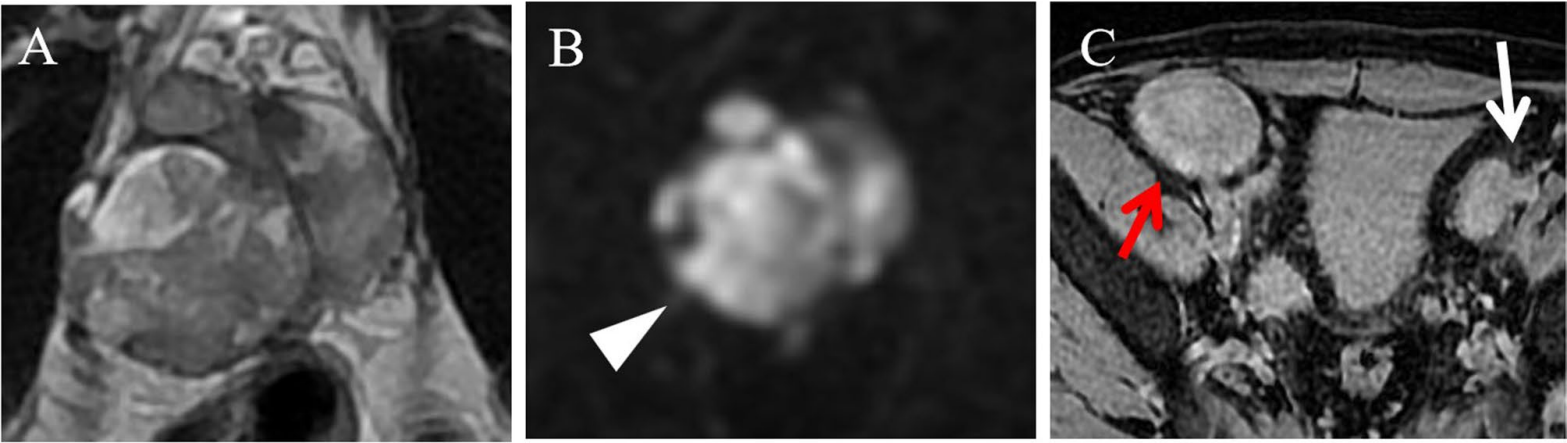
MP-MRI indicates an enlargement of the lesion scope compared to the previous scan (**A**), with pronounced diffusion restriction (white triangle, **B**). Enlarged lymph nodes are observed adjacent to the left iliac vessels (white arrow, **C**). The red arrow points to the transplanted kidney

The patient revisited our hospital owing to pelvic pain 4 months ago. Blood tests revealed a WBC count of 6.3 × 10⁹/L, CRP level of 5.9 mg/L, a tPSA level of 7.29 ng/mL, and a SCr level of 144 µmol/L. Urine culture indicated a positive result for candida glabrata. Multiparametric magnetic resonance imaging (Mp-MRI) demonstrated an enlargement of the lesion size compared to previous images (Fig. 3A, B), along with the presence of several newly enlarged lymph nodes beside the bilateral iliac vessels (Fig. 3C).

**Fig. 4:**
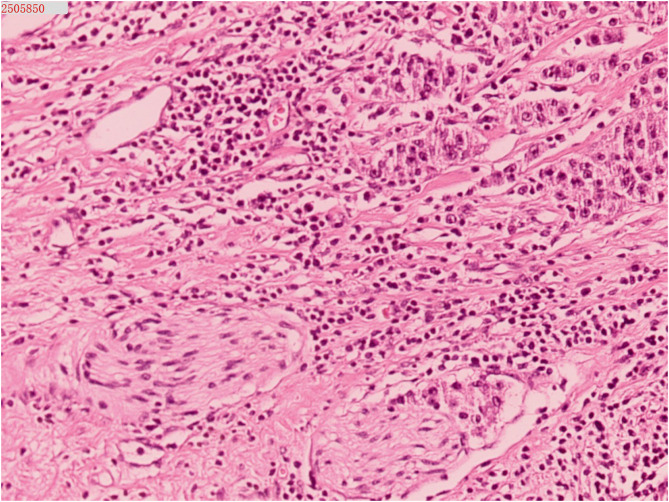
Histopathology: Poorly differentiated prostatic adenocarcinoma (HE×200)

Subsequently, the patient underwent an ultrasound-guided transperineal prostate biopsy. The pathological findings indicated a poorly differentiated malignant tumor. To further evaluate the potential for distant metastasis, the following staging investigations were completed: Whole-body bone scan and chest CT revealed no significant abnormalities, while abdominal CT scan demonstrated enlarged lymph nodes within the pelvic cavity. One week later, the patient underwent laparoscopic radical prostatectomy (RP). During the surgery, it was noted that the prostate was significantly enlarged, with severe adhesion to the bladder neck. Additionally, a swollen lymph node measuring approximately 2 cm was identified beside the left iliac vessel, and a left iliac vessel and obturator lymph node dissection was performed. The final pathological result revealed a poorly differentiated prostate cancer with necrosis, involving 90% of the total prostate volume (Fig. 4). Extraprostatic extension : (+), surgical margin of the specimen (prostatic dissection surface) : (+). Intravascular tumor emboli: (+), perineural invasion : (+). No cancer metastasis was observed in the prefatty tissue of the prostate: (0/2), metastasis was found in the lymph node adjacent to the left iliac vessel: (1/1). The pTNM staging (AJCC 8th) is pT4N1Mx. Discharge Diagnoses: (1)Prostate cancer, (2) Renal allograft dysfunction.

After the surgery, endocrine therapy was administered using leuprorelin acetate sustained-release microspheres for injection (11.25 mg). Due to the patient’s development of stress urinary incontinence, mirabegron sustained-release tablets (50 mg) were added as an adjunctive treatment. Additionally, the patient was instructed to perform Kegel exercises to strengthen pelvic floor muscle function. Simultaneously, maintain the original immunosuppressive treatment regimen. The patient exhibited favorable postoperative recovery and has maintained regular follow-up visits. To date, no signs of recurrence have been identified.

## Discussion

By conducting a comprehensive analysis of the clinical data provided in this case study, we have attributed the causes of misdiagnosis to the following factors. Initially, the patient’s clinical symptoms, including low-grade fever, perineal heaviness, and urinary tract infections, were highly similar to those of a PA. Against the backdrop of a young patient’s long-term use of immunosuppressants, clinicians considered a high likelihood of infection. Moreover, after the initial treatment, the patient reported an improvement in symptoms, which seemed to confirm the diagnosis of a PA. Secondly, the patient’s laboratory results lacked typicality. Inflammatory markers such as CRP and WBC count were partially elevated. In two PSA tests, the tPSA levels fell within the gray zone (4–10 ng/mL), while the ratios of free PSA (fPSA) to tPSA remained normal. Additionally, the urine culture yielded positive results, which, to a certain extent, interfered with clinical judgment. Thirdly, imaging examinations failed to fully exert their functions. The lesion exhibited atypical hyperintensity on T2WI. It is hypothesized that this imaging finding may be correlated with intralesional hemorrhage. During the initial MRI examination, the diffusion restriction of the lesion was not prominent. Ultrasound examination is closely associated with the operator’s experience and subjectivity, which serves as one of the primary causes of misdiagnosis in this case.

It is noteworthy that the use of immunosuppressants after kidney transplantation has been proven to increase the incidence of malignancies and infections. Among RTRs, urogenital malignancies rank as the third most common malignancy, following newly-emerged skin malignancies and post-transplant lymphoproliferative disorders. PCa, as one of the most prevalent types, has an incidence rate of 0.72–3.1%, which is significantly higher than that of age-matched controls in the general population [3, 4]. In addition, the age of onset of PCa detected in RTRs is approximately 62.3 years old, which is earlier than the 70 years old in the general population. To date, there is no standard PCa screening protocol for RTRs, nor have relevant guidelines for PSA testing been established. Based on the hemodynamic characteristic that t-PSA is mainly metabolized by the liver, it is speculated that t-PSA may serve as a relatively reliable tumor marker for RTRs. According to relevant literature reports, when the t-PSA level ranges from 4 to 10 ng/mL, the prevalence rate of PCa is only 17%, if the t-PSA level exceeds 10 ng/mL, the risk increases to approximately 50% [5]. Given that the renal graft in the iliac fossa may affect radiotherapy planning, RP remains the preferred treatment modality for localized PCa. A relevant study involving 9 transplant centers in Germany revealed that the incidence of postoperative complications in the RTR group was higher (14.5%) compared to that in the non- RTR cohort (1–8%) [3]. Dat et al., through a retrospective study of 525 patients, found that the overall survival rates of RTRs who underwent surgery and radiotherapy showed no significant difference from those of the general population [6].

 In clinical settings, when encountering cases with high-risk factors for PCa yet atypical clinical symptoms and imaging manifestations, it is imperative to re-evaluate and contemplate conducting PCa screening. To facilitate the achievement of early and accurate diagnosis of PCa, effective information can be obtained through the following clinical approaches.

1. The role of DRE in the diagnosis of PCa: As a simple and cost-effective physical examination method, DRE has an overall accuracy rate of 63.45% [7]. Thus, DRE serves as a convenient screening method for PCa, necessitating further investigations to confirm or rule out PCa.

2. The role of biomarkers in the diagnosis of PCa: PSA is currently the most widely used biomarker for PCa diagnosis. However, its limited specificity and the resulting overtreatment remain its drawbacks, which compel people to search for biomarkers with heightened predictive potential for PCa. The 4-Kallikrein score (4Kscore) combines the measurements of four biomarkers with clinical data (including age, DRE results, and history of negative biopsies) to predict the risk of aggressive tumors [8].The prostate health index (PHI) integrates three major indicators, namely p2PSA, tPSA and fPSA. It maintains high sensitivity while increasing the specificity to 42% [9]. When circulating tumor cells (CTC) are integrated with PSA testing or other relevant assays, the predictive accuracy can reach 90% [10]. In conclusion, the integration of multiple biomarkers is an indispensable component for enhancing the diagnostic accuracy of PCa.

3. The role of MRI in the diagnosis of PCa: As the gold-standard imaging technique for staging and localizing PCa lesions, Mp-MRI, which comprises high-resolution T2WI, DWI, and dynamic contrast-enhanced (DCE) sequences, demonstrates an overall sensitivity of 0.74 and specificity of 0.88 [11]. This technique spares 27% of patients from undergoing prostate biopsy [12]. The Prostate Imaging Reporting and Data System (PI-RADS) based on Mp-MRI demonstrates a relatively high diagnostic accuracy for clinically significant prostate cancer (csPCa). In recent years, Bp-MRI which includes both T2WI and DWI sequences, has been demonstrated to be non-inferior to Mp-MRI, and it offers the advantages of shorter scanning time while eliminating the risks associated with contrast agents [13]. However, a clear consensus has not yet been reached.

4. The role of ultrasound in the diagnosis of PCa: Transrectal ultrasonography (TRUS), as a commonly used imaging modality in urology, now plays a guiding role during prostate biopsy and treatment. CEUS can serve as an effective complementary tool for Bp-MRI in the diagnosis of PCa. The combined use of these two modalities contributes to improving diagnostic accuracy, particularly in patients with renal insufficiency or those allergic to gadolinium-based contrast agents [14]. Ultrasound elastography (UE) quantitatively reflects the stiffness of focal tissues, demonstrating higher accuracy in detecting aggressive lesions [15]. However, ultrasound examination is susceptible to the operator’s proficiency and subjective factors, which may introduce bias into the examination results.

5. The role of PET/CT in the diagnosis of PCa: Prostate-specific membrane antigen (PSMA) PET/CT reflects the physiological metabolism of tissues. Compared with traditional imaging modalities, it exhibits greater specificity in the detection of pelvic lymph node metastases or distant metastases [16, 17]. Previous research has indicated that the maximum standardized uptake value (SUVmax) on PSMA PET/CT is significantly correlated with PSMA expression levels, Gleason scores, and tumor aggressiveness. Consequently, it can serve as a crucial biomarker for distinguishing csPCa from benign prostatic diseases [18]. However, due to the high affinity of the ribs and pelvic bones for radiolabeled tracers, which can lead to false-positive results, additional examinations are required to clarify the status of metastasis.

6. The role of Artificial Intelligence (AI) in the diagnosis of PCa: With the continuous advancement of AI within the field of modern medicine, the application of AI in the diagnosis, differentiation, and evaluation of PCa demonstrates both feasibility and applicability [19, 20]. However, when compared with traditional inspection approaches, the accuracy and interpretability of this method still necessitate extensive clinical trials for validation.

Since the advent of antibiotics, the incidence of PA has been continuously declining, accounting for approximately 0.5% of all urinary system diseases, with a higher prevalence among patients with diabetes and those with compromised immune function [21]. Symptoms of PA overlap with those of other urinary system diseases. Its characteristic sign is tenderness upon palpation in the prostatic region during DRE. However, studies have indicated that only 52.2% of PA patients exhibit positive DRE findings [22]. It is challenging to differentiate between PA and PCa based solely on medical history and physical examination. Mp-MRI plays a pivotal role in the diagnostic process of PA. The MRI manifestations of PA are largely dependent on the structures within the abscess wall and the abscess cavity. DCE-MRI imaging can reveal significant enhancement of the abscess wall with no enhancement in the abscess cavity, whereas entire lesion of PCa exhibits pronounced and homogeneous enhancement. Additionally, the pathological differences between PCa and PA manifest as signal alterations on DWI and apparent diffusion coefficient (ADC) maps. Specifically, the degree of diffusion restriction is lower in PCa than in PA, and the ADC values of PCa are higher than those of PA [23]. Therefore, DWI sequence exhibits high sensitivity in detecting minute abscesses. In this case, the enhanced appearance of the region with restricted diffusion on DWI is clearly inconsistent with the typical imaging manifestations of PA. Furthermore, PCa tissue generally presents as uniformly hypointense on T2WI, while PA often shows hyperintensity due to intracavitary liquefactive necrosis. The aforementioned imaging features can also serve as distinguishing points between the two diseases.

## Conclusion

The misdiagnosis of PCa is influenced by multiple factors, including insufficient specificity of PCa-related biomarker testing, atypical imaging findings, patients’ comorbidities, and insufficient understanding among clinicians about PCa. Therefore, it is essential to integrate multiple examination techniques to establish a multimodal diagnostic system, and strengthen training for clinicians on complex PCa cases, thereby improving diagnostic accuracy.

## Supplementary Information

Supplementary Material 1

## Data Availability

All data generated or analysed during this study are included in this published article.

## Abbreviations

PCa: Prostate cancer
PA: Prostatic abscess
RTR: Renal transplant recipient
Mp-MRI: Multiparametric magnetic resonance imaging
PSA: Prostate specific antigen
