# Supplementary material for: Prostate cancer misdiagnosed as prostatic abscess: case report and literature review
Source: BMC Urol. 2025 Nov 18;25:289. doi: 10.1186/s12894-025-01982-6 (PMC12625306; doi:10.1186/s12894-025-01982-6)
Supplement: Supplementary file 1 — Supplementary Material 1 [file 12894_2025_1982_MOESM1_ESM.pdf]

CARE case report guidelines

CARE Checklist of information to include when writing a case report

(cc) BY-NC-ND

Topic	Item	Checklist item description	Reported on Line
Title	1	The diagnosis or intervention of primary focus followed by the words "case report"	Line 1-2
Key Words	2	2 to 5 key words that identify diagnoses or interventions in this case report, including "case report"	Line 27
Abstract (no references)	3a	Introduction: What is unique about this case and what does it add to the scientific literature?	Line 16
	3b	Main symptoms and/or important clinical findings	Line 17
	3c	The main diagnoses, therapeutic interventions, and outcomes	Line 18
	3d	Conclusion—What is the main "take-away" lesson(s) from this case?	Line 21-25
Introduction	4	One or two paragraphs summarizing why this case is unique (**may include references**)	Line 43-47
Patient Information	5a	De-identified patient specific information.	Line 51,53-54
	5b	Primary concerns and symptoms of the patient.	Line 52
	5c	Medical, family, and psycho-social history including relevant genetic information	Line 53
	5d	Relevant past interventions with outcomes	Line 54
Clinical Findings	6	Describe significant physical examination (PE) and important clinical findings.	Line 51-52,54-56
Timeline	7	Historical and current information from this episode of care organized as a timeline	Line 51-52,80,95,100
Diagnostic Assessment	8a	Diagnostic testing (such as PE, laboratory testing, imaging, surveys).	Line 55-61,80-86,95-100
	8b	Diagnostic challenges (such as access to testing, financial, or cultural)	Line 130-145
	8c	Diagnosis (including other diagnoses considered)	Line 110-111
	8d	Prognosis (such as staging in oncology) where applicable	N/A
Therapeutic Intervention	9a	Types of therapeutic intervention (such as pharmacologic, surgical, preventive, self-care)	Line 100-101,113-117
	9b	Administration of therapeutic intervention (such as dosage, strength, duration)	Line 113-118
	9c	Changes in therapeutic intervention (with rationale)	N/A
Follow-up and Outcomes	10a	Clinician and patient-assessed outcomes (if available)	Line 118-120
	10b	Important follow-up diagnostic and other test results	N/A
	10c	Intervention adherence and tolerability (How was this assessed?)	Line 118-119
	10d	Adverse and unanticipated events	N/A
Discussion	11a	A scientific discussion of the strengths AND limitations associated with this case report	Line 128-145
	11b	Discussion of the relevant medical literature **with references**.	Line 147-255
	11c	The scientific rationale for any conclusions (including assessment of possible causes)	Line 148-160,179-190,240-254
	11d	The primary "take-away" lessons of this case report (without references) in a one paragraph conclusion	Line 258-263
Patient Perspective	12	The patient should share their perspective in one to two paragraphs on the treatment(s) they received	N/A
Informed Consent	13	Did the patient give informed consent? Please provide if requested	Yes ☑ No ☐
